# Simulation of head and neck cancer oxygenation and doubling time in a 4D cellular model with angiogenesis

**DOI:** 10.1038/s41598-017-11444-1

**Published:** 2017-09-08

**Authors:** Jake C. Forster, Michael J. J. Douglass, Wendy M. Harriss-Phillips, Eva Bezak

**Affiliations:** 1 Department of Physics, University of Adelaide, North Terrace, Adelaide, South Australia 5005 Australia; 2 Department of Medical Physics, Royal Adelaide Hospital, North Terrace, Adelaide, South Australia 5000 Australia; 3 Sansom Institute for Health Research and the School of Health Sciences, University of South Australia, Adelaide, South Australia Australia

## Abstract

Tumor oxygenation has been correlated with treatment outcome for radiotherapy. In this work, the dependence of tumor oxygenation on tumor vascularity and blood oxygenation was determined quantitatively in a 4D stochastic computational model of head and neck squamous cell carcinoma (HNSCC) tumor growth and angiogenesis. Additionally, the impacts of the tumor oxygenation and the cancer stem cell (CSC) symmetric division probability on the tumor volume doubling time and the proportion of CSCs in the tumor were also quantified. Clinically relevant vascularities and blood oxygenations for HNSCC yielded tumor oxygenations in agreement with clinical data for HNSCC. The doubling time varied by a factor of 3 from well oxygenated tumors to the most severely hypoxic tumors of HNSCC. To obtain the doubling times and CSC proportions clinically observed in HNSCC, the model predicts a CSC symmetric division probability of approximately 2% before treatment. To obtain the doubling times clinically observed during treatment when accelerated repopulation is occurring, the model predicts a CSC symmetric division probability of approximately 50%, which also results in CSC proportions of 30–35% during this time.

## Introduction

While tumors are typically more vascularized than normal tissue, hypoxia will still arise in many tumors due to heterogeneity in the vascularity and depleted levels of blood oxygenation that occur when blood moves sluggishly through constricted and malformed vessels^1, 2^. Radiotherapy is a primary treatment modality for head and neck squamous cell carcinoma (HNSCC) and tumor oxygenation has been correlated with treatment outcome^3–5^. Another key factor influencing the treatment outcome is the rate of tumor regrowth during treatment. Radiotherapy is typically delivered over several weeks (an example of a conventional fractionation schedule for the treatment of HNSCC with X-rays is 2 Gy fractions, 5 days/week over 6 weeks), and after a certain time (the “kick-off time”) the tumor initiates accelerated repopulation^6–8^. One of the key mechanisms responsible for accelerated repopulation is reportedly an increase in the symmetric division of cancer stem cells (CSCs). CSCs make up only a small proportion of the tumor cells^9^, but each has the potential to regenerate the tumor and must be inactivated to achieve 100% local tumor control probability.

In previous work, a computational model was developed that simulates HNSCC tumor growth^10^. This is a 4D cellular model that includes the simulation of tumor angiogenesis. In the first part of the current work, this model was used to quantitatively map tumor properties, such as vascularity and blood oxygenation, to tumor oxygenation descriptors, such as the proportion of hypoxic cells, the mean cellular pO_2_ and the necrotic volume. By constraining the vascularity and blood oxygenation to values that have been clinically observed in HNSCC, values of tumor oxygenation descriptors were obtained for HNSCC and compared with clinical data.

In the second part of the work presented here, the effect of tumor oxygenation on the tumor volume doubling time for HNSCC was quantitatively assessed. The effect of the CSC symmetric division probability on the doubling time was also explored. The CSC symmetric division probability also affects the proportion of CSCs in the tumor and this relationship was investigated. Finally, doubling times and CSC proportions were compared with clinical data for HNSCC. While tumor irradiation was not simulated, tumor growth kinetics during accelerated repopulation were obtained by increasing the CSC symmetric division probability.

## Methods

### The tumor growth model

**Figure 1:**
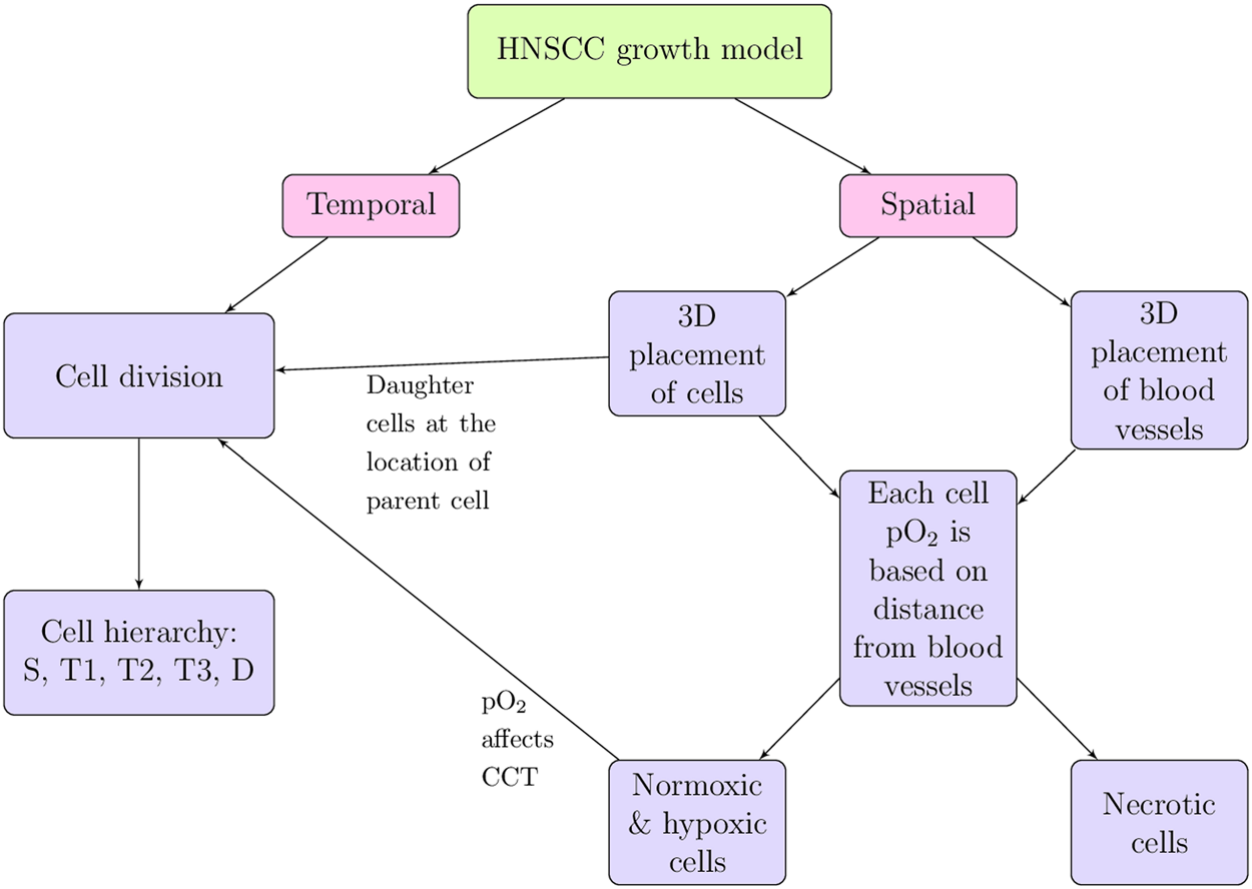
Main features of the HNSCC tumor growth model. Adapted with permission from ref. 10.

**Figure 2:**
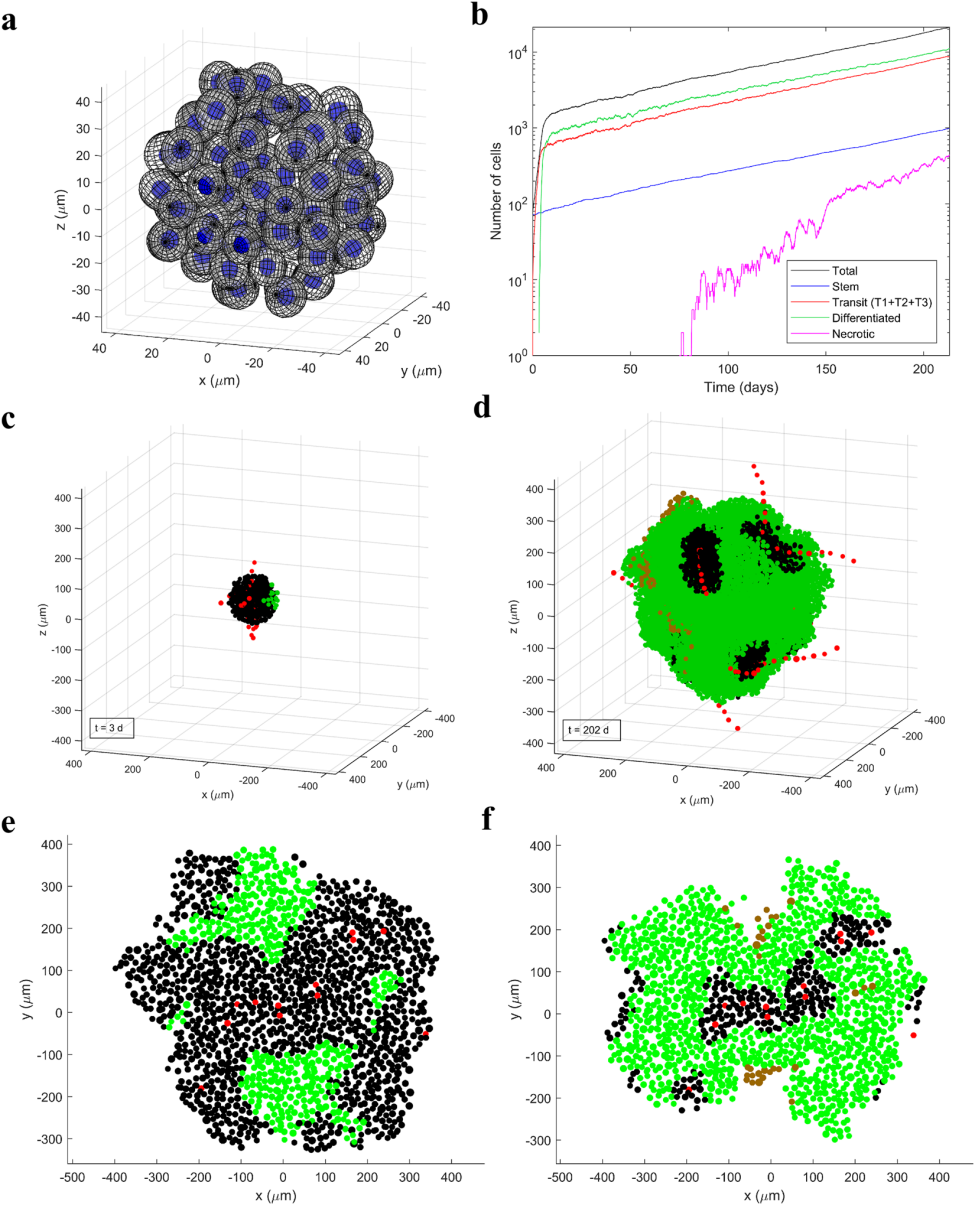
HNSCC tumor growth model. (**a**) Tumor cells are modeled as non-overlapping ellipsoids in randomized positions in 3D. (**b**) Cell kinetics for the different types of cells in an example simulation. (**c**,**d**) The tumor in the example simulation after 3 days and 202 days of growth. Vessel units are shown in red, normoxic cells in black, hypoxic cells (pO_2_ < 10 mmHg) in green and necrotic cells in brown. Vessel units “string” together to form whole vessels that undergo branching in a chaotic fashion. Tumor cells close to vessels are normoxic, cells further from vessels are hypoxic and cells pushed further than *ND* from a vessel become necrotic. This example simulation started with approximately 70 CSCs and ended with *RVV* = 0.4%, using *p*_0_ = 40 mmHg, *ND* = 180 µm and CSC symmetric division probability = 2%. (**e**,**f**) Sections of tumors with the same vasculature structure but different blood oxygenation (via *p*_0_ and *ND*) ((**e**) *p*_0_ = 60 mmHg and *ND* = 220 µm; (**f**) *p*_0_ = 30 mmHg and *ND* = 120 µm). (**e**,**f**) were adapted with permission from ref. 10.

Simulations of HNSCC tumor growth were performed using a computational model that was developed in-house using Matlab (version R2017a, The MathWorks, Inc.) and has been previously described^10^. The flow chart in Fig. 1 outlines the spatial and temporal features of the model and how they are related. Briefly, each tumor cell is modeled as an ellipsoid and packed into randomized positions in 3D space without overlap (Fig. 2a). The tumor grows over time by cell division, wherein a cell upon reaching the end of its cell cycle time (CCT) divides into two daughter cells, consequently pushing neighbouring cells outward towards the tumor periphery. A hierarchy of cell types is simulated, including CSCs, three generations of transit cells (T1-3) and differentiated cells (Fig. 2b). The probability for CSCs to undergo symmetric division (i.e., divide into two CSCs as opposed to one CSC and one transit cell) is set by the user. The sloughing of differentiated cells, which is characteristic of epithelial tissue, is also simulated.

Angiogenesis is modeled reflecting a connected and chaotic tumor vasculature that grows out with the cells (Fig. 2c,d), with blood vessels represented by consecutive discrete vessel units. Tumors can be grown with different vascularities which are quantified by the relative vascular volume, *RVV*. Cellular pO_2_ is modeled dynamically as a function of distance from the nearest vessel using a diffusion equation (Table 1), with key parameters being the blood oxygenation, *p*_0_, and the distance from vessels to the onset of necrosis (the necrosis distance, *ND*). The tumor vascularity (*RVV*) and the blood oxygenation (*p*_0_ and *ND*) affect the amount of hypoxia in the tumor (Fig. 2e,f). Hypoxic cells have longer CCTs and cells that become necrotic are gradually resorbed by the tumor. Table 1 summarises the main parameters of the model and their values for HNSCC.

**Table 1:** Tumor growth model input parameters and values for HNSCC.

Input parameter	Values	Type	References
Cell (and blood vessel) diameter	14–20 µm	Distribution	11, 36, 37
*RVV*	2–10%	Single value	11, 12
Oxygen tension	$p(r)={p}_{0}\frac{N{D}^{2}}{{{R}_{0}}^{2}}(2\,\mathrm{ln}(\frac{ND}{r})-1+\frac{{r}^{2}}{N{D}^{2}})$ where${{R}_{0}}^{2}=N{D}^{2}(2\,\mathrm{ln}(\frac{ND}{a})-1)$, *a* = 10 µm	Distribution	38, 39
*p*_*0*_	20–100 mmHg	Single value	11, 13
*ND*	80–300 µm	Single value	14, 15
CCT under normoxia	33 ± 5.9 h (Gaussian)	Distribution	40, 41
CCT adjustment factor with hypoxia	$y({{\rm{pO}}}_{2})=1+1.8{e}^{-0.2{{\rm{pO}}}_{2}}$	Distribution	40, 42, 43
Hypoxia-induced quiescence	pO_2_ < 1 mmHg	Single value	44–46
Necrotic cell resorption time	3–6 days depending on local necrotic volume	Distribution	47, 48
CSC symmetric division probability	~2% pre-treatment, possibly >50% during accelerated repopulation	Single value	6–8, 30, 40
Differentiated cell loss frequency	80%	Single value	40, 41

Simulations entail the following. First, a unique 3D mesh of non-overlapping cell/vessel unit positions is generated using Monte Carlo methods. The cell density reached is 2 × 10^8^ cells/cm^3^. A connected network of blood vessels is then generated. As a result, a selection of the mesh positions are designated as vessel unit positions. A unique vasculature is generated each time using Monte Carlo methods. The vasculature is chaotic and tortuous, representative of tumor vasculature^1, 2^. As the tumor grows larger, the vasculature grows out by activating more of the vessel unit positions. Mesh positions that are not designated as vessel unit positions are cell positions, meaning tumor cells may occupy them during tumor growth simulation.

Once a blood vessel network has been generated and prior to tumor growth simulation, the oxygen tension at each cell position is determined and used to calculate the CCT. Cells push one another around when a cell divides, a differentiated cell is lost or a necrotic cell is resorbed. When a cell changes position, it retains its age (the time since it last divided), but its CCT changes to the CCT at its new position. When the age of the cell equals its position dependent CCT, it divides. When a cell divides, it pushes a neighbouring cell outward towards the tumor periphery, causing a chain of cell movement outward, making room for the additional daughter cell. Thus, one daughter cell occupies the position where the parent cell used to be, and the other daughter cell occupies an adjacent position.

The daughter cells are always one generation more differentiated than the parent cell (CSC → T1 → T2 → T3 → differentiated), except in the case of CSC symmetric division. A differentiated cell loss frequency of 80% is simulated to model the natural cell death of these cells, i.e., apoptosis. When a cell becomes differentiated, after a time equal to the CCT, there is an 80% likelihood that the differentiated cell is removed from the tumor. If it is not removed, it remains for another period of time equal to the CCT, then there is again an 80% likelihood that it is removed, and so on. When a differentiated cell is removed, there is a chain of cell movement inward to fill the vacant position. The same occurs when a necrotic cell is resorbed from the tumor, which occurs when its age reaches the necrotic cell resorption time (Table 1).

For a more in-depth description of the computational model methods, please refer to ref. 10.

### Study of tumor oxygenation

The HNSCC tumor model was used in this work to quantify how the vascularity (*RVV*) and the blood oxygenation (*p*_0_ and *ND*) affect the tumor oxygenation. Noting that HNSCC exhibit *RVV* from 2–10%^11, 12^, *p*_0_ from 20–100 mmHg^11, 13^ and *ND* from 80–300 µm^14, 15^, three combinations of (*p*_0_, *ND*) were considered, namely (20 mmHg, 80 µm), (40 mmHg, 180 µm) and (100 mmHg, 300 µm), depicting scenarios of poor, moderate and high blood oxygenation, respectively. In each case, the model input parameter *RVV*_0_ (which would be equal to the tumor *RVV* if the tumor grew with spherical symmetry, but due to preferential growth along the vessels, *RVV* ends up larger than *RVV*_0_) was varied from 0–10% in 1% increments (for the (20 mmHg, 80 µm) combination, *RVV*_0_ values of 0.25% and 0.5% were also used), yielding values of tumor *RVV* from 0–16%. The tumor *RVV* was determined at the end of the growth simulation as the ratio of the number of vessel units to the number of living cells + necrotic cells + vessel units (×100%). Tumor growth simulations began with approximately 70 CSCs and ended with a final tumor diameter of 1 mm (10^4^–10^5^ cells). A CSC symmetric division probability of 50% was used in the tumor oxygenation study for fast computations, since this affects the doubling time and CSC proportion but does not greatly affect the tumor oxygenation.

The tumor oxygenation at the end of the growth simulation was evaluated using several different descriptors. The hypoxic proportions HP_10_, HP_5_, HP_2.5_ and HP_1_ were determined, which were the proportions of living cells with pO_2_ <10, 5, 2.5 and 1 mmHg, respectively. The mean and median cellular pO_2_ in living cells were also calculated. The volume proportion of necrosis (necrotic volume) was evaluated as the ratio of the number of necrotic cells to the number of living cells + necrotic cells + vessel units (×100%).

### Study of tumor growth rate and CSC proportion

The HNSCC tumor model was then used to explore the effects of tumor oxygenation and CSC symmetric division on the doubling time and the CSC proportion. The doubling time, *T*_*d*_, in the final days (in “tumor time”) of the tumor growth simulation was evaluated as follows. Let *N(t)* denote the number of living and necrotic cells in the tumor at time *t*. Then the average slope, *k*, of the curve ln*N* vs *t* in the final few days of the simulation was used to calculate the final doubling time according to:

$${T}_{d}=\,\mathrm{ln}\,2/k$$

The effect of tumor hypoxia and necrosis on doubling time was observed in the simulations from the tumor oxygenation study. Since these simulations all used a CSC symmetric division probability of 50%, the relative variation in the doubling time was reported.

To investigate the effects of the CSC symmetric division probability on the doubling time and the CSC proportion, CSC symmetric division probabilities of 2%, 5%, 10%, 25%, 50%, 75% and 100% were simulated for the two extremes of HNSCC tumor oxygenation. The most oxygenated case was *RVV* = 10%, *p*_0_ = 100 mmHg and *ND* = 300 µm and the most hypoxic case was *RVV* = 2%, *p*_0_ = 20 mmHg and *ND* = 80 µm. In order to achieve approximately these *RVV*s, the model parameter *RVV*_0_ was set to 8.2% and 0.75% respectively. Again, the simulations began with approximately 70 CSCs and ended with a final tumor diameter of 1 mm. Three simulations (n = 3) were conducted for each value of CSC symmetric division probability for both well oxygenated and severely hypoxic cases (with the exception of the severely hypoxic case with CSC symmetric division probability 10%, for which n = 4 was used). The CSC proportion was calculated as the ratio of the number of CSCs to the number of living cells (×100%). The doubling times and CSC proportions were plotted using the mean value of the 3 (or 4) simulation runs and with error bars corresponding to the standard error of the mean (SEM). Prism (version 7, GraphPad Software, Inc.) was used to determine whether statistical significance had been reached.

### Equipment

Simulations were performed on the Phoenix cluster at the University of Adelaide^16^ using as many as 12 cores and 10 GB of RAM.

### Data availability

The data that support the finding of this study are available from the corresponding author upon reasonable request.

### Code availability

The code used to analyze the data is available from the corresponding author upon reasonable request. The code used to perform tumor growth simulations has not been made publicly available at this time.

## Results

### The effects of tumor vascularity and blood oxygenation on tumor oxygenation

**Figure 3:**
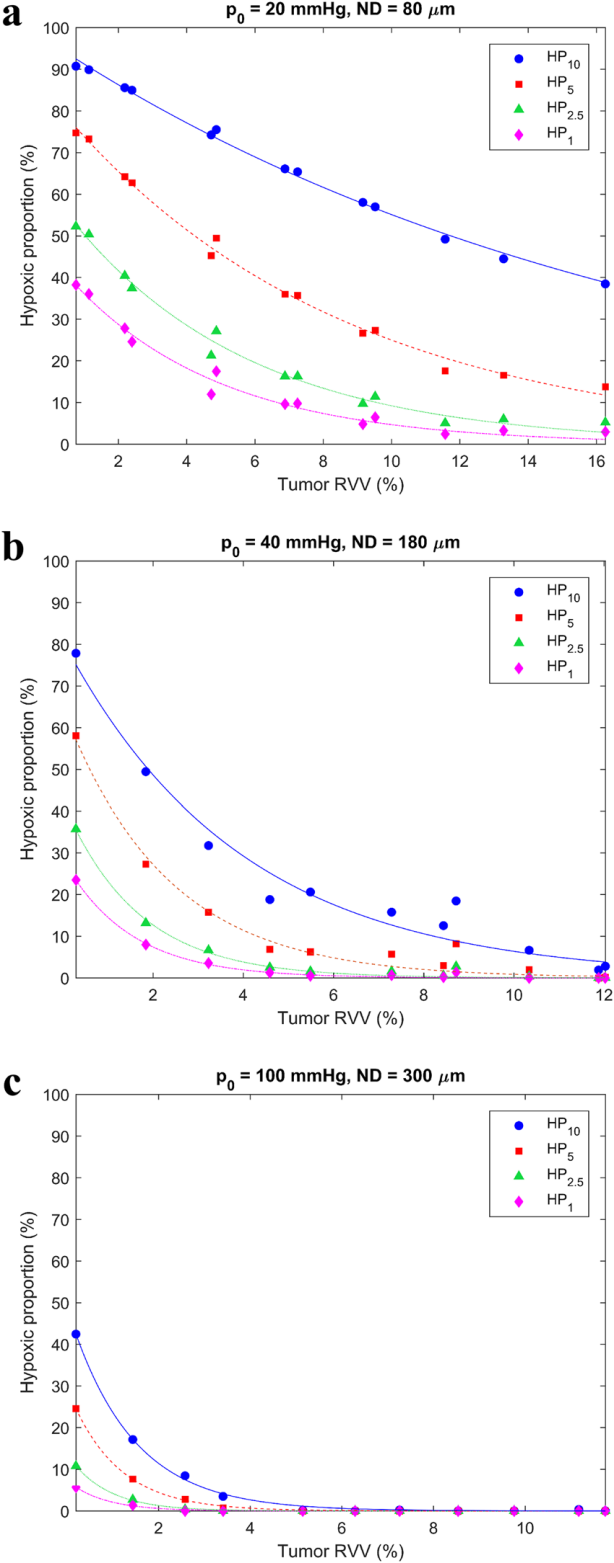
Variation of hypoxic proportions with tumor vascularity for (**a**) poor blood oxygenation (*p*_0_ = 20 mmHg and *ND* = 80 µm), (**b**) moderate blood oxygenation (*p*_0_ = 40 mmHg and *ND* = 180 µm) and (**c**) high blood oxygenation (*p*_0_ = 100 mmHg and *ND* = 300 µm).

**Figure 4:**
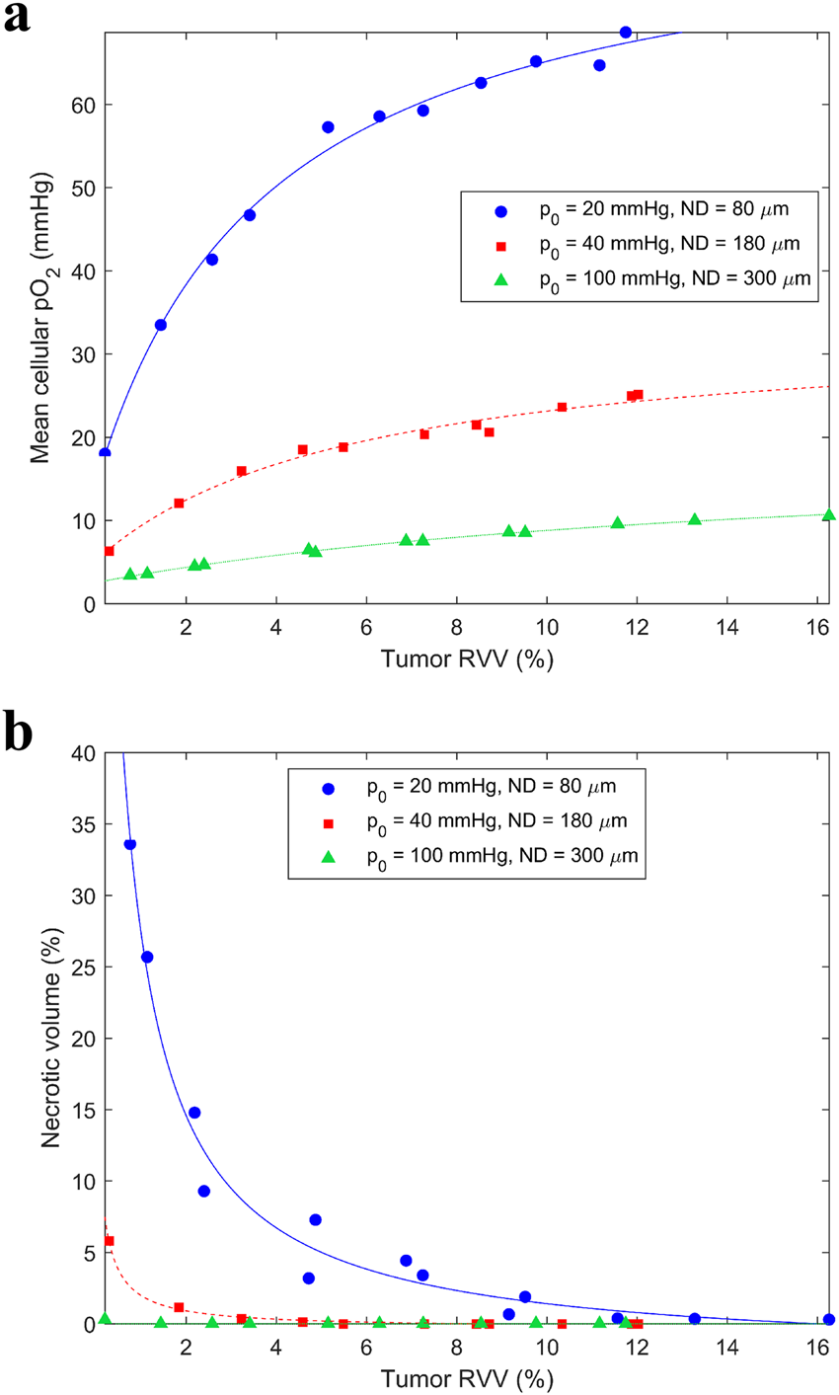
Variation of (**a**) mean cellular pO_2_ and (**b**) necrotic volume with tumor vascularity for poor, moderate and high blood oxygenation.

With increasing tumor vascularity (*RVV*) and increasing blood oxygenation (*p*_0_ and *ND*), the hypoxic proportions and necrotic volume decreased, while the mean and median cellular pO_2_ increased (Figs 3 and 4). According to clinical data, HNSCC exhibit *RVV* from 2–10%^11, 12^, *p*_0_ from 20–100 mmHg^11, 13^ and *ND* from 80–300 µm^14, 15^. With these constraints, the tumor growth model predicted values of HP_1_ from 0–29%, HP_2.5_ from 0–42%, HP_5_ from 0–65%, HP_10_ from 0–86%, mean cellular pO_2_ from 4.4–65.2 mmHg, median cellular pO_2_ from 2.9–67.5 mmHg and necrotic volume from 0–15% for HNSCC.

For poor blood oxygenation (*p*_0_ = 20 mmHg and *ND* = 80 µm), there was necrosis present even at 10% *RVV*. Note that the same hypoxic proportion could arise from different combinations of *RVV*, *p*_0_ and *ND*. For example, (*RVV*, *p*_0_, *ND*) combinations of (11.8%, 20 mmHg, 80 µm), (2.7%, 40 mmHg, 180 µm) and (0.4%, 100 mmHg, 300 µm) each yielded a HP_5_ of 20%.

### The effects of hypoxia and CSC symmetric division on the tumor growth rate and the CSC proportion

**Figure 5:**
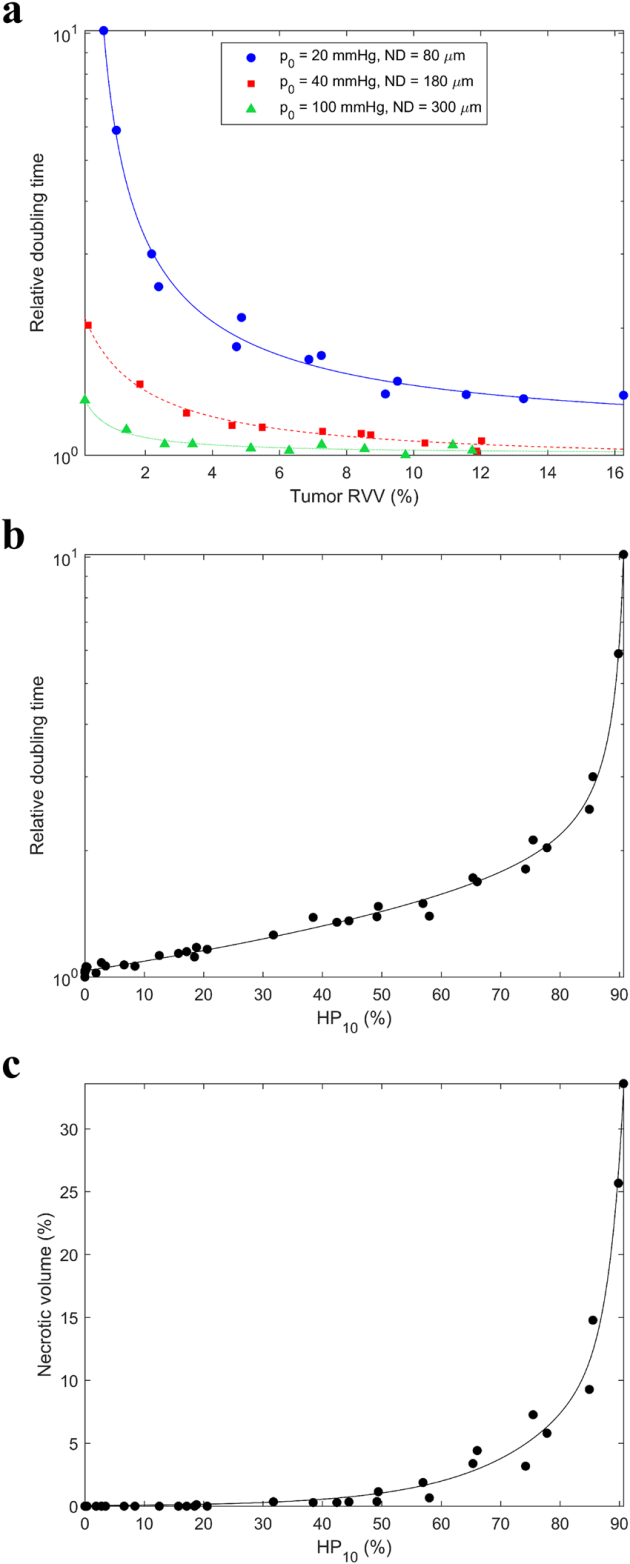
Relative variation in the doubling time with (**a**) tumor vascularity and (**b**) HP_10_. (**c**) Variation in the necrotic volume with HP_10_.

The doubling time decreased with increasing tumor vascularity (*RVV*) and increasing blood oxygenation (*p*_0_ and *ND*) (Fig. 5a). For HNSCC (*RVV* = 2–10%, *p*_0_ = 20–100 mmHg, *ND* = 80–300 µm), the doubling time increased by a factor of 3 from well oxygenated tumors to the most hypoxic tumors. The doubling time was considerably affected, even without the presence of necrosis, by low cellular pO_2_ effects such as increased CCTs and cell quiescence (Fig. 5b,c).

**Figure 6:**
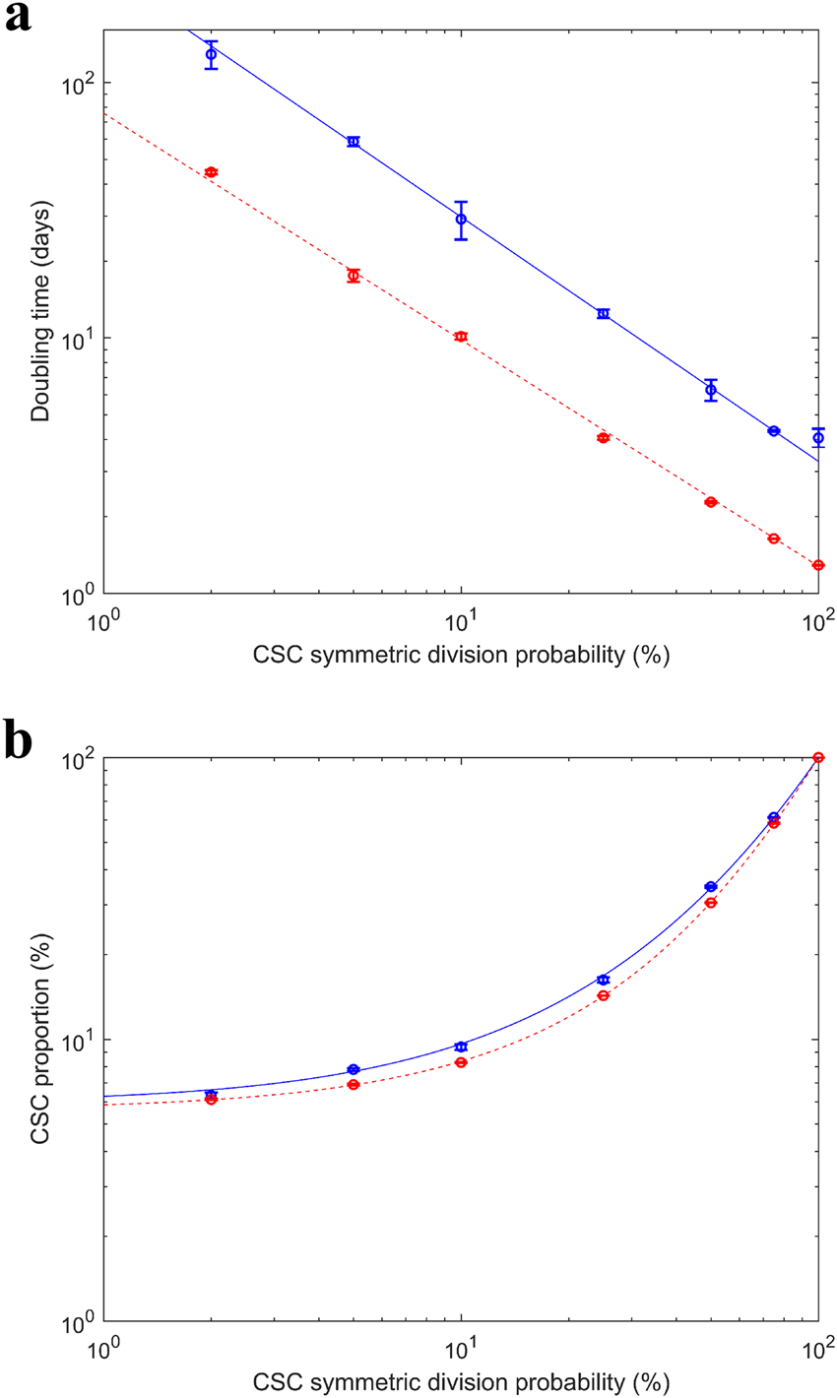
Variation of (**a**) doubling time and (**b**) CSC proportion with CSC symmetric division probability for severely hypoxic tumors (mean ± SD *RVV* = 2.2 ± 0.2% (n = 22), *p*_0_ = 20 mmHg, *ND* = 80 μm) (blue) and well oxygenated tumors (mean ± SD *RVV* = 10.5 ± 0.6% (n = 21), *p*_*0*_ = 100 mmHg, *ND* = 300 μm) (red) of HNSCC.

The doubling time decreased with increasing probability of CSC symmetric division probability (Fig. 6a). The mean doubling time was 2.6–3.3 times larger for the most hypoxic tumors than for well oxygenated tumors of HNSCC across all values of CSC symmetric division probability. The difference in doubling time between severely hypoxic and well oxygenated conditions was significant (p-value < 0.05 using unpaired t-test with Welch’s correction) at every value of CSC symmetric division probability.

The CSC proportion increased with CSC symmetric division probability (Fig. 6b). The mean CSC proportion was 1–1.14 times larger for the most hypoxic tumors than for well oxygenated tumors across all values of CSC symmetric division probability. The difference in CSC proportion between severely hypoxic and well oxygenated conditions was significant (p-value <0.05 using unpaired t-test with Welch’s correction) at every value of CSC symmetric division probability except 2%.

A CSC symmetric division probability of 2% yielded a mean ± SEM doubling time of 44.5 ± 0.8 days (n = 3) for well oxygenated tumors and 129 ± 16 days (n = 3) for the most hypoxic tumors. The CSC proportion was approximately 6% in each case (6.11 ± 0.01% (n = 3) and 6.3 ± 0.2% (n = 3) respectively). An increase in the CSC symmetric division probability to 50% yielded a doubling time of 2.28 ± 0.03 days (n = 3) for well oxygenated tumors and 6.3 ± 0.6 days (n = 3) for the most hypoxic tumors. The CSC proportions were 30.49 ± 0.06% (n = 3) and 34.8 ± 0.3% (n = 3) respectively.

## Discussion

Several studies have reported pO_2_ measurements in human HNSCC using invasive polarographic needle electrodes. The results from some of these studies are collated in Table 2. Since accessing a tissue block from the HNSCC primary tumor can be difficult, these studies often took measurements from sufficiently large lymph node metastases originating from a primary HNSCC. The table also lists measurements of necrotic volume in HNSCCs, as assessed by CT scan or MRI. The results from the current study are included at the bottom for comparison.

**Table 2:** Clinical data for tissue pO_2_ and necrotic volume in human HNSCC.

	Tumor site	Median pO_2_ (mmHg) in the tumor	Mean pO_2_ (mmHg) in the tumor	HP_2.5_ (%)	HP_5_ (%)	HP_10_ (%)	Necrotic volume (%)
King *et al*.^49^	Metastastic cervical nodes from HNSCC	—	—	—	—	—	mean ± SD 19.09 ± 13.94 (n = 106)
Kong *et al*.^50^	Primary HNSCC	mean 14.0 (n = 82)	—	—	—	—	—
Gagel *et al*.^51^	lymph node metastases from HNSCC	mean ± SD 12.5 ± 10.3; range 0.1–41.1 (n = 38)	mean ± SD 17.6 ± 7.3; range 8.8–36.0 (n = 38)	mean ± SD 29.3 ± 18.4; range 0.0–58.5 (n = 38)	mean ± SD 38.4 ± 18.1; range 7.0–73.6 (n = 38)	mean ± SD 48.9 ± 18.2; range 13.0–78.7 (n = 38)	—
Nordsmark *et al*.^3^	Neck node metastases from HNSCC or primary HNSCC	median 9; range 0–62 (n = 397)	—	median 19; range 0–97 (n = 397)	median 38; range 0–100 (n = 397)	—	—
Kuhnt *et al*.^52^	Primary HNSCC	—	—	—	—	—	mean ± SD 18 ± 30 (n = 51)*
Gagel *et al*.^53^	neck lymph node metastases from HNSCC	mean 10.7; 95% CI of mean 5.2–16.1; range 0.4–22.4 (n = 16)	mean 16.3; 95% CI of mean 12.1–20.5; range 9.0–27.4 (n = 16)	mean 35.9; 95% CI of mean 24.1–47.6; range 0.5–58.1 (n = 16)	mean 44.3; 95% CI of mean 34.0–54.5; range 27.6–66.5 (n = 16)	mean 52.5; 95% CI of mean 42.2–62.9; range 33.7–77.5 (n = 16)	—
Terris *et al*.^54^	cervical lymph node metastases from HNSCC	—	mean ± SD 20.8 ± 13.7 (n = 50)*	mean ± SD 20.8 ± 25.7 (n = 42)	—	—	mean ± SD 14.5 ± 11.2 (n = 42)
Brizel *et al*.^55^	Primary HNSCC or cervical lymph node from HNSCC	mean 4.5; range 0–60 (n = 63)*	—	—	—	—	—
Brizel *et al*.^5^	Primary HNSCC or neck node metastases from HNSCC	mean 11.2; range 0.4–60 (n = 28)	—	—	—	—	—
Nordsmark *et al*.^4^	Lymph node metastases from HNSCC (n = 34) or primary HNSCC (n = 1)	mean ± SD 14.7 ± 10.8; median 14; range 1–35 (n = 35)	—	mean ± SD 22 ± 24; median 15; range 0–95 (n = 35)	mean ± SD 35 ± 29; median 29; range 0–100 (n = 35)	—	—
The current work		range 2.9–67.5	range 4.4–65.2	range 0–42	range 0–65	range 0–86	range 0–15

*Mean ± SD of two or more sub-groups were combined with appropriate error propagation.

The values of median pO_2_, mean pO_2_, HP_2.5_, HP_5_ and HP_10_ produced by the tumor growth model using *RVV* from 2–10%, *p*_0_ from 20–100 mmHg and *ND* from 80–300 µm are in line with these clinical measurements. This assists in model validation since these values for *RVV*, *p*_0_ and *ND* are based on clinical data for HNSCC. The clinical tumor oxygenation data overall indicate well oxygenated tumors are rare and lower values of *RVV*, *p*_0_ and *ND* are typical.

The clinical studies sometimes reported large values of necrotic volume outside the range produced by the tumor growth model. This is likely because the clinical studies observed macroscopic regions of necrosis. Macroscopic necrosis occurs when tumors become large and whole macroscopic regions of the tumor lose blood supply. The tumor growth model only produced necrosis at the microscopic scale (between distant blood vessels) in the sub-clinical sized tumors used in this study.

Pre-treatment doubling times of HNSCC have been obtained in studies that measured tumor growth while patients waited for treatment. Jensen *et al*.^17^ found that in the time between diagnostic scan (MR or CT) and treatment planning CT scan (median 28 days, range 5–95 days), the median doubling time was 99 days (range 15 to > 234 days) for 61 patients with HNSCC. Waaijer *et al*.^18^ found that in the time between diagnostic and treatment planning CT scans (mean 34 days), the mean doubling time was 96 days (range 21–256 days) in 13 patients with oropharyngeal SCC. Murphy *et al*.^19^ found that in the time between diagnostic (MRI or CT) and planning or interval CT scan (median 35 days, range 8–314 days), the median doubling time was 94 days (range 16–6931 days) in 85 oropharyngeal SCC. These average clinically measured pre-treatment doubling times are similar to those produced by the presented tumor growth model under moderately hypoxic conditions and with a CSC symmetric division probability of approximately 2% (recall the doubling times obtained with the tumor model using 2% CSC symmetric division averaged 45 days for well oxygenated tumors and 130 days for severely hypoxic tumors).

In the current work, a CSC symmetric division probability of 2% yielded a proportion of CSCs in the tumor of approximately 6% for all HNSCC tumor oxygenation levels. Methods have been established for identifying CSCs in HNSCC. For example, cells that express markers such as ALDH1, CD133 and CD44 exhibit CSC-like properties, while others do not^9, 20–24^. Cells that efficiently efflux Hoechst 33342 dye, termed side-population (SP) cells, are also CSC-like^25, 26^. Chinn *et al*.^27^ reported a mean CD44^high^ content of 10.8% (range 0–84.5%) for 40 patient-derived primary HNSCCs. In 10 human oral SCC tissue samples, Zhang *et al*.^28^ found CD133^+^ content of 1–3%. Lu *et al*.^29^ identified approximately 2.1% SP cells in 7 human primary HNSCC samples, and all SP cells were also CD133^+^. The CSC proportion of 6% obtained by the tumor growth model using 2% CSC symmetric division, is close to these clinical estimates for HNSCC pre-treatment and results from other models (e.g. 5.9% from Marcu & Marcu^30^).

Tumors respond to treatment by undergoing accelerated repopulation. In an analysis of 5 clinical trials containing a total of 2653 patients, Pedicini *et al*.^31^ using an analytical/graphical method arrived at a best estimate of 3.5 days (95% CI 3.1–3.9 days) for the doubling time of HNSCC during radiotherapy. The loss of asymmetric division by CSCs is believed to be a key mechanism behind accelerated repopulation^6–8, 30, 32, 33^. In the tumor growth model, a CSC symmetric division probability of 50% yielded doubling times from 2.3 to 6.1 days, depending on the tumor oxygenation, which are in line with the estimate by Pedicini *et al*. A 50% CSC symmetric division probability yielded CSC proportions from 30–35% in the current work. To the authors’ knowledge, there are no clinical studies in the literature that measured the CSC proportion in HNSCC in patients during accelerated repopulation. In the model by Marcu & Marcu^30^, the CSC proportions obtained were higher than in the current work for the same CSC symmetric division probability. For example, in their work, 10%, 20% and 30% symmetric division yielded 25%, 35% and 45% CSCs, respectively (recall in the current work, 10% and 25% symmetric division yielded approximately 9% and 15% CSCs, respectively). Conversely, the CSC proportions were slightly lower in the HYP-RT model by Harriss-Phillips *et al*.^33^ than in the current work. In that model, 30% symmetric division yielded just 10% CSCs. Most *in vitro* studies of various cancer types show a 3–5 times increase in CSCs post single irradiation^24^.

## Conclusion

The current work established how the tumor oxygenation varies with vascularity and blood oxygenation, how the doubling time varies with tumor oxygenation and CSC symmetric division probability, and how the CSC proportion varies with CSC symmetric division probability in a 4D cellular model of HNSCC tumor growth. The doubling time varied by a factor of ~3 from well oxygenated tumors to the most severely hypoxic tumors of HNSCC. A CSC symmetric division probability of 2% yielded clinically relevant doubling times and CSC proportions for HNSCC before treatment, while a value of 50% produced the doubling times observed in the clinic for HNSCC undergoing accelerated repopulation. This 50% probability yielded CSC proportions from 30–35%.

In future work, the tumor growth model will be extended to a radiotherapy simulation tool for both low and high LET beams. The cellular geometry will be imported into Geant4^34^ and irradiated in Monte Carlo track structure simulations. Radiolysis will be simulated along the particle tracks. Ionisation events and generated ^•^OH species will be clustered in the cell nuclei to predict the complexity and extent of DNA damage to each cell. The cellular pO_2_ will affect how efficiently ^•^OH attack to the base of DNA is translated to strand breakage^35^. Irradiation will be simulated in fractions separated by time intervals, during which the tumor growth model will translate DNA damage to cell death while also regrowing the tumor.
